# Fiber and Electrical Field Alignment Increases BDNF Expression in SH-SY5Y Cells following Electrical Stimulation

**DOI:** 10.3390/ph16020138

**Published:** 2023-01-17

**Authors:** Quy-Susan Huynh, R. M. Damian Holsinger

**Affiliations:** 1 Laboratory of Molecular Neuroscience and Dementia, School of Medical Sciences, Faculty of Medicine and Health, The University of Sydney, Camperdown, NSW 2050, Australia; 2 Neuroscience, School of Medical Sciences, Faculty of Medicine and Health, The University of Sydney, Sydney, NSW 2006, Australia

**Keywords:** electrospinning, electrical stimulation, neurotrophic factors

## Abstract

The limited expression of neurotrophic factors that can be included in neural tissue engineering scaffolds is insufficient for sustained neural regeneration. A localized and sustained method of introducing neurotrophic factors is required. We describe our attempt at inducing neuroblastoma cells to express trophic factors following electrical stimulation. Human SH-SY5Y neuroblastoma cells, cultured on polycaprolactone electrospun nanofibers, were electrically stimulated using a 100 mV/mm electric field. Nuclear morphology and brain-derived neurotrophic factor (BDNF) expression were analyzed. Cells were classified based on the type of fiber orientation and the alignment of these fibers in relation to the electric field. Nuclear deformation was mainly influenced by fiber orientation rather than the electrical field. Similarly, fiber orientation also induced BDNF expression. Although electrical field alone had no significant effect on BDNF expression, combining fiber orientation with electrical field resulted in BDNF expression in cells that grew on electrospun fibers that were aligned perpendicular to the electrical field.

## 1. Introduction

The peripheral nervous system is capable of regeneration depending on the severity of the damage [1]. Optimal regenerative outcomes are more likely to occur when axons have a conduit to support and guide their regeneration, such as an intact connective sheath [2]. Conduits made from biomaterials are often utilized where peripheral nerve damage is severe and has resulted in transection and gaps of up to 30 mm [3,4]. These conduits are often constructed from biopolymers and are designed to degrade after tissue regeneration has occurred [4]. Autografts are the current gold standard treatment [5] but lead to secondary site donor morbidity [6], which can lead to decrease in function or lead to irritation/pain [7]. Other treatment options include allografts or xenografts, which have resulted in higher levels of immune responses and have not performed as well as autografts [8]. With improvements in the process of decellularization, allografts such as AxoGen have improved and overcome some of these issues [9]. Progress in decellularization technology has similarly improved xenografts [10].

Advancements in biomimetic scaffolds including those made from synthetic materials have demonstrated that the physical and mechanical properties of tissue engineering scaffolds can have a profound effect on cells and gene expression [11]. Mesenchymal stem cells preferably differentiated towards a bone lineage on stiffer substrates compared to softer substrates where these cells differentiate towards a neural lineage [12]. Mechanical forces effect cells through the cytoskeleton and its nucleus via cellular and nuclear mechanotransduction. Such forces transmitted through the cytoskeleton can affect cell shape and activities such as migration [13], whilst those transmitted through cytoskeletal structures to the nucleus exert effects on cellular activity through changes in nuclear shape and ultimately the expression of specific genes [14].

Neurotrophic factors have regenerative effects on cells of the nervous system. Nerve growth factor (NGF) and brain-derived neurotrophic factor (BDNF) are known for enhancing growth and survival of neurons [15,16]. The major hurdle of delivering exogenous neurotrophic factors directly to target tissues would be the lack of control over neurotrophic factor diffusion away from the target tissue. Furthermore, there is currently no established method for sustained delivery of neurotrophic factors to nervous system tissue. Temporal and spatial issues could be potentially addressed by employing biomaterials that are infused with and gradually elute neurotrophic factors [17]. However, the issue of a discrete or limited amount of neurotrophic factor release from the eluting material still presents an unresolved issue. Withdrawal of neurotrophic factor release following regeneration has been shown to lead to further degeneration [18] and is another unresolved issue facing treatment.

Cells of the nervous system are considered excitable, leading to an assumption that neural tissue would respond to electrical stimulation (ES). Electrical signals are naturally present in the body, even during embryonic development [19]. EFs have been shown to develop during wound formation and are believed to play an electrotactic role in wound healing [20]. The use of ES following nerve injury has resulted in better recovery outcomes and quicker recovery times [21,22]. ES has been shown to effect neural stem cell fate [23] as well increase the expression of neurotrophic factors in certain neuronal populations [24,25].

Research into the microenvironment of cells has revealed the importance of both biochemical and physical cues to cell function and tissue regeneration. Emulating many components of the microenvironment of cells may therefore improve tissue regeneration. Electrospun nanofibrous scaffolds have been well researched for their ability to mimic the nanotopography of the extracellular matrix [26,27]. These scaffolds have also played a role in stem cell differentiation [27,28], gene expression in neuronal cells [29], and can even influence immune responses [30]. Aligned and non-aligned fiber patterns have been known to effect cellular behavior. Chew and colleagues demonstrated that Schwann cells seeded onto aligned electrospun fibers expressed higher levels of genes related to maturing Schwann cells compared to those seeded on randomly aligned fibers [29]. 

Enhanced gene expression may be reflective of mechanotransduction properties transferred to the nucleus and may in turn promote the expression of specific genes. Combined with mechanical cues from the electrospun scaffolds, ES could be employed to enhance the expression of neurotrophic factors to aid nerve tissue regeneration. This is a potential solution that can produce localized and sustainable sources of neurotrophic factor. In this study, we aimed to investigate how ES delivered along aligned or non-aligned nanofibrous scaffolds could modulate the expression of BDNF in SH-SY5Y human neuroblastoma cells.

## 2. Results

Human SH-SY5Y neuroblastoma cells are a well-characterized cell line that is used for research of the nervous system. Here, we grew SH-SY5Y cells on both aligned and non-aligned electrospun fibers to determine whether growth on these scaffolds changed cellular morphology and neurotrophic factor expression. 

### 2.1. Analysis

**Figure 1:**
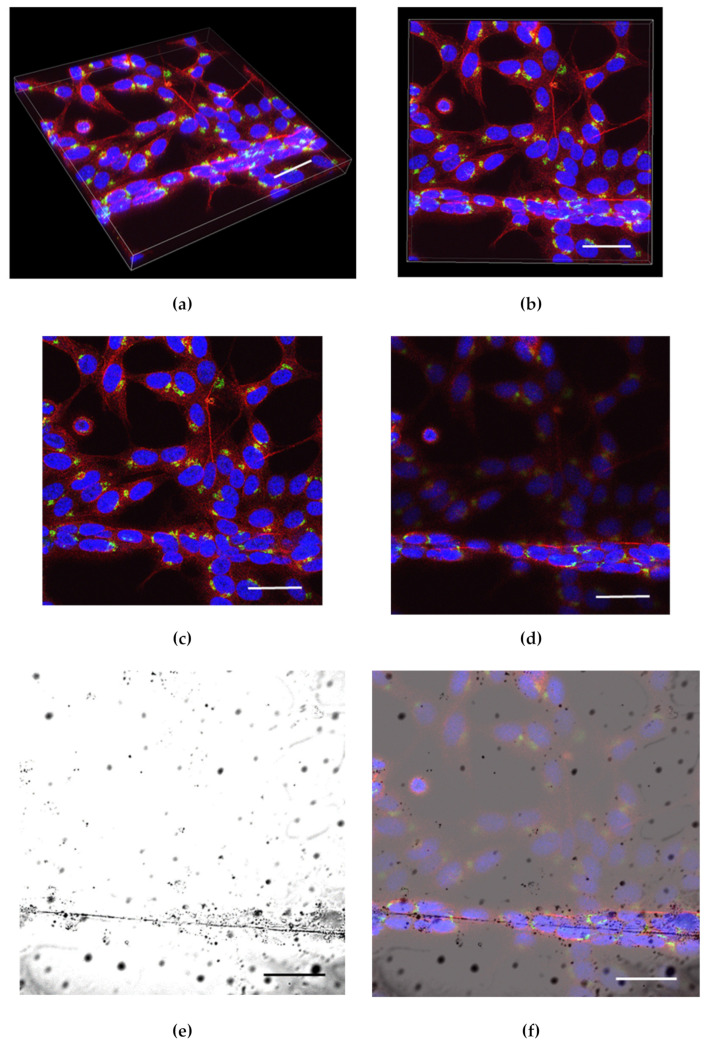
Volume of Z stack images: (**a**) Isometric view (depth 16.80 µm); (**b**) top view. Slice used to analyze cells classified as (**c**) flat and (**d**) aligned. Both slices obtained from the volume in (**a**) and (**b**). Overlay of fluorescent image on transmitted image to demonstrate the growth of cells on fiber (**e**). Transmitted light image and (**f**) transmitted light image overlayed with RGB image. The overlayed image verifies the presence of cells growing on fibers. BDNF (green), β-tubulin (red), and DAPI (blue) (scale bar 40 µm).

Z-stack images were obtained using 60× water lens (Plan Apo 60xA/1.2 WI; Tokyo, Japan) on a Nikon C2 Confocal microscope (Tokyo, Japan). Z slices (0.8 µm thick) were selected from stack images based on slices that had the brightest fluorescence for cells on glass slips and fibers (Figure 1a–d).

**Figure 2:**
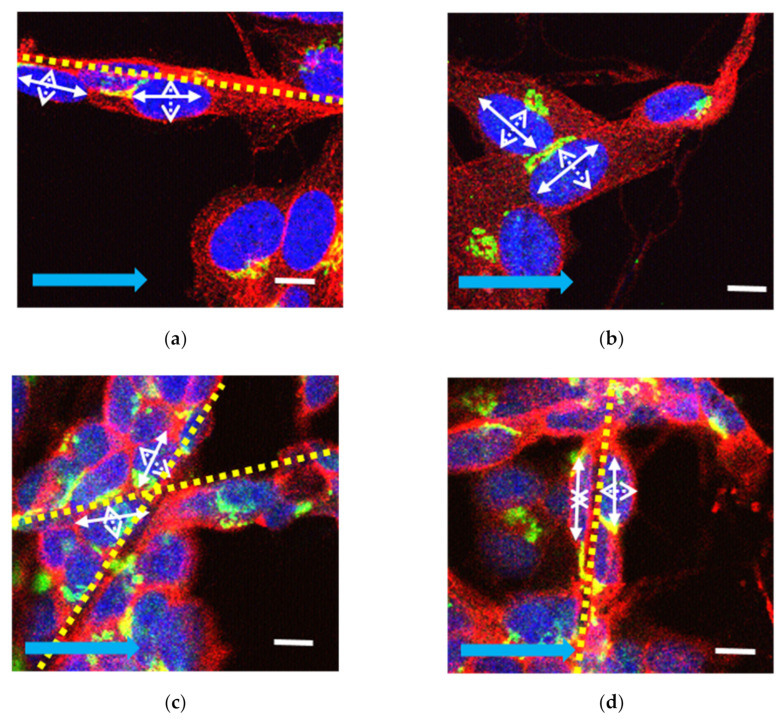
Examples of cells classified as aligned (**a**), flat (**b**), non-aligned (**c**), and perpendicular (**d**). Aligned cells lie on electrospun fibers also aligned with the EF direction (**a**), cells grown in between where fibers cross (**c**), and cells grown on aligned fibers orientated perpendicular to the direction of the EF (**d**). Major (long) and minor (short) axes of human SH-SY5Y cell nuclei are illustrated using a solid white arrow to represent the major (long) axis of the nucleus and a dashed white arrow, the minor (short) axis. Yellow dashed lines represent electrospun fibers. Solid blue arrow represents the direction of the EF. BDNF (green), β-tubulin (red), and DAPI (blue) (scale bar 10 µm).

To confirm cell attachment to the scaffold, transmitted images were obtained and laid over the fluorescence Z-stack (Figure 1e,f). The slices were segmented using ImageJ version 1.8.0_172 (ImageJ, National Institutes of Health, Bethesda, MD, USA). Cells were selected based on the orientation of the cell growing on the fibrous scaffolds as well as their orientation in relation to the direction of the EF (Figure 2).

**Figure 3:**
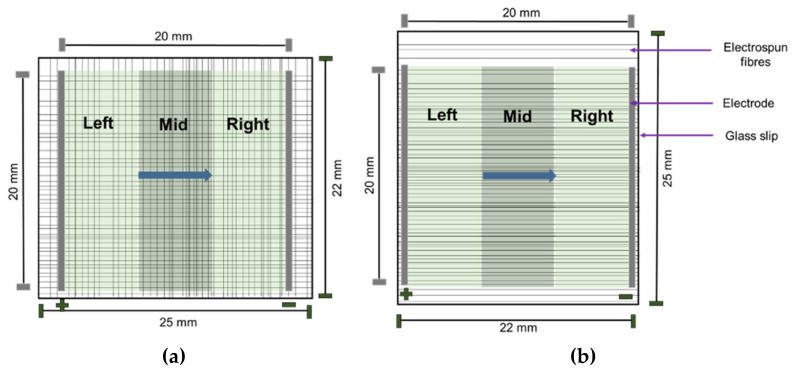
Schematic illustrating non-aligned fibers (**a**) and aligned fibers (**b**). The blue arrow represents the direction of the EF. The green area represents area of the EF (20 mm × 20 mm) on glass slips (25 mm × 22 mm). Cell locations are classified as left, mid, and right depending on the position of the cells in relation to the EF as depicted above.

We employed a 4-tier classification system based on the manner in which cells grew: (i) cells that grew on plain glass coverslips were classified as ‘flat’; (ii) cells growing on aligned fibers that were also aligned with the direction of the EF were classified as ‘aligned’; (iii) cells growing on aligned fibers that were perpendicular to the direction of the EF were classified as ‘perpendicular’; and iv) cells found at corners where perpendicular fibers intersected were labelled as ‘non-aligned’. Cells were also classified based on their location in relation to the EF (Figure 3). The images were subsequently added to the open-source software CellProfiler [31] for quantification of BDNF expression and characterization of nuclear morphology. Statistical analyses were conducted using Microsoft Excel and SPSS version 25 (IBM) for ANOVA and post-hoc Tukey analysis. For uneven sample sizes, Tukey–Kramer was automatically implemented [32]. R version 3.6.3 was used for statistical summaries and to plot the data.

### 2.2. Fiber Orientation Influences Nuclear Morphology

**Figure 4:**
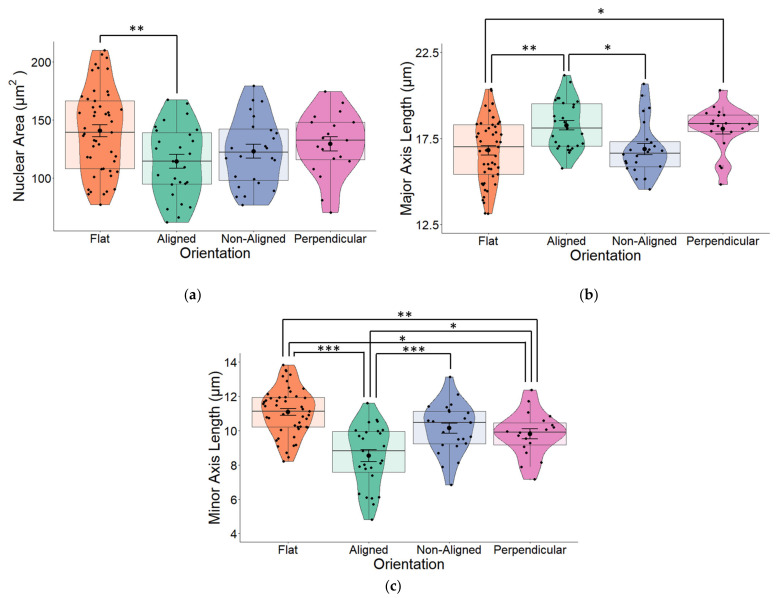
(**a**) The nuclear area of human SH-SY5Y cells grown on aligned or non-aligned scaffolds without application of an EF. Cells were grown either on plain glass coverslips (control) or on electrospun (ES) glass coverslips. Electrospinning was performed to produce either aligned or non-aligned fibers. Cells growing on aligned fibers that were perpendicular to the direction of the electrical field (EF) were classified as ‘perpendicular’. Cells were fluorescently stained with DAPI to identify the nucleus. Measurement of the surface area of the nuclei revealed substantial variations based on the medium on which it was grown. Whilst cells grown on scaffolds all displayed smaller nuclear surface areas (one-way ANOVA *p* = 0.009), those grown on aligned scaffolds displayed significantly smaller surface areas (Tukey post-hoc *p* = 0.006) compared to control cells grown on plain glass coverslips. (**b**) Quantification of nuclear length along the major (long) axis of non-stimulated SH-SY5Y cells grown on glass coverslips that were either plain (flat) or coated with electrospun PCL fibers in either an aligned or non-aligned manner. One-way ANOVA *p* < 0.001. Measurement of the cell nuclei along the major (long) axis revealed that cells grown on aligned fibers that were also aligned with the EF and cells grown on aligned fibers perpendicular to the EF had significantly longer nuclei compared to those grown on either flat (plain coverslips–control) or non-aligned fibers (post-hoc Tukey *p* = 0.002 and *p* = 0.031 respectively). The major axis of non-aligned cells was significantly smaller than aligned cells (*p* = 0.02). (**c**) Quantification of nuclear length along the minor (short) axis of non-stimulated SH-SY5Y cells. One-way ANOVA *p* < 0.001. Measurement of the cell nuclei along the minor (short) axis revealed that all cells grown on electrospun fibers had significantly narrower/short nuclei compared to those grown on flat (plain coverslips–control). Post-hoc Tukey * *p* < 0.05, ** *p* < 0.01, *** *p* < 0.001 Flat *n* = 49, aligned *n* = 27, non-aligned *n* = 24, perpendicular *n* = 19.

We observed that fiber orientation of the scaffold significantly altered the area of the cell nucleus (one-way ANOVA *p* = 0.009) as well as the major (long) and minor (short) axes (both *p* < 0.001) of the nucleus. Cells grown on glass coverslips (flat) that were used as controls for all our experiments had the largest surface area (Figure 4a). Although all cells grown on fibers had reduced nuclear areas, the nucleus of cells grown on perpendicular fibers and especially aligned fibers took on a more elongated morphology, which was evident from the major (long) axis (Figure 4b). The greatest changes in nuclear morphology resulting from fiber orientation were reflected in the minor (short) axes of cells were significant changes (one-way ANOVA *p* < 0.001; Figure 4c), were observed across all three fiber orientations compared to the nuclei of cells that were grown on flat surfaces. Cells grown on aligned fibers had the smallest minor axis (*p* < 0.001).

### 2.3. BDNF Expression Is Influenced by Fiber Orientation

**Figure 5:**
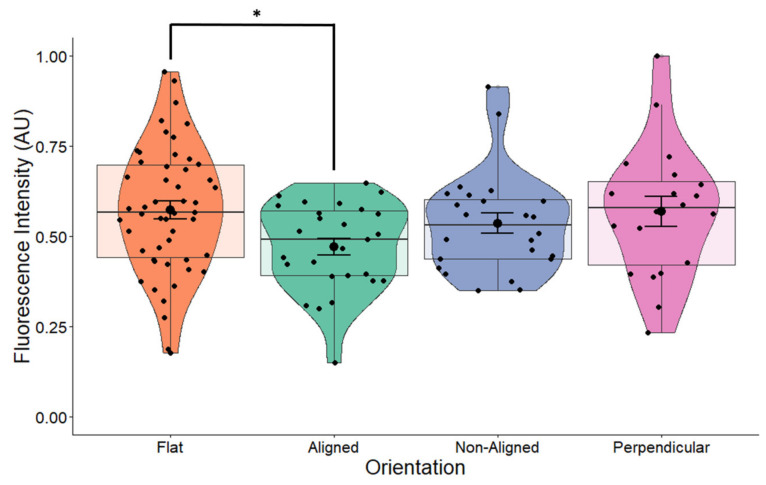
BDNF expression in non-stimulated SH-SY5Y cells grown on scaffolds with different orientations. BDNF expression was measured in fluorescently labeled cells using confocal microscopy. BDNF expression levels were significantly decreased (*p* = 0.042) in cells grown on aligned scaffolds. * *p* < 0.05. Flat *n* = 49, aligned *n* = 27, non-aligned *n* = 24, perpendicular *n* = 19.

Changes in cellular and nuclear morphology are known to regulate transcription and translation and are mediated through proteins in the cell membrane and the nuclear pore. To determine whether the morphological changes observed in the cell nucleus would have an effect on neurotrophic factor expression, we examined the expression of BDNF. Overall, fiber orientation had a marginal effect on the expression of BDNF in cells grown on scaffolds (*p* = 0.055) (Figure 5). Cells grown on aligned fibers had significantly lower expression of BDNF (*p* = 0.042) compared to control cells. Expression of BDNF in cells grown on non-aligned and perpendicularly aligned cells did not appear to change compared to the control. Comparing these results to those shown in Figure 4a, our results indicate that changes in nuclear morphology led to minimal changes in BDNF protein expression.

### 2.4. Effect of EF on Nuclear Morphology

**Figure 6:**
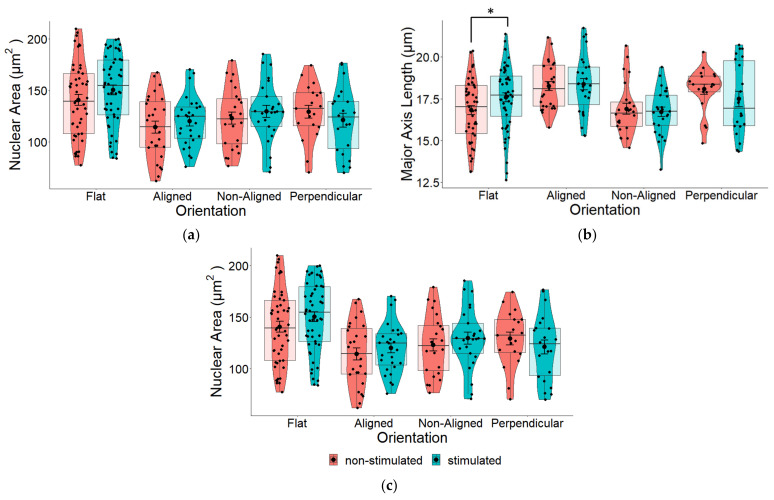
(**a**) Pair-wise comparison of the nuclear area of SH-SY5Y cells grown on aligned or non-aligned scaffolds with (ES—stimulated) and without (Control—non-stimulated) EF. The effect of EF on nuclear area was not significant: one-way ANOVA for the comparison between stimulated and non-stimulated cells on flat substrates *p* = 0.174, aligned *p* = 0.433, non-aligned *p* = 0.441, and perpendicular *p* = 0.377. (**b**) Pair-wise comparison of nuclear surface area along the major (long) axis of SH-SY5Y cells with (ES) and without (Control) EF. One-way ANOVA for the comparison between stimulated and non-stimulated cells: *p* = 0.046 (flat), *p* = 0.795 (aligned), *p* = 0.567 (non-aligned), and *p* = 0.313 (perpendicular). * *p* < 0.05. (**c**) Pair-wise comparison of nuclear surface area along the minor (short) axis of SH-SY5Y cells with (ES) and without (Control) EF. One-way ANOVA for the comparison between stimulated and non-stimulated cells: *p* = 0.344 (flat), *p* = 0.225 (aligned), *p* = 0.382 (non-aligned), *p* = 0.454 (perpendicular). Flat *n* = 101, aligned *n* = 54, non-aligned *n* = 49, perpendicular *n* = 41.

**Figure 7:**
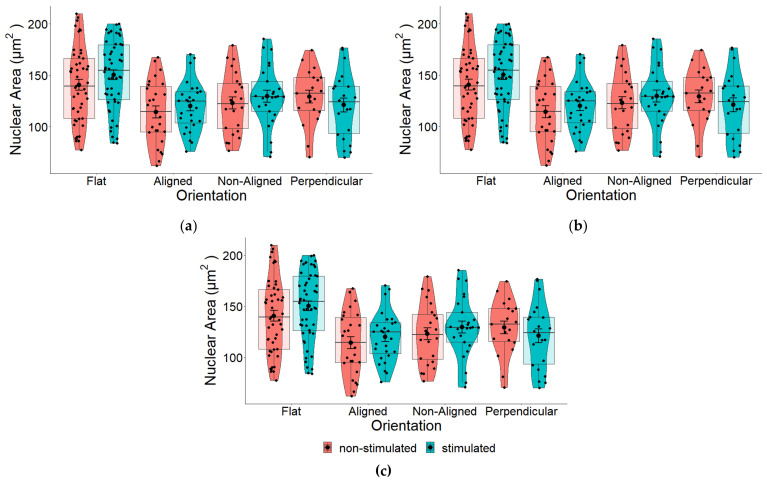
Pair-wise comparison of (**a**) total nuclear surface area, (**b**) major and (**c**) minor axes of SH-SY5Y cells following ES based on location (Figure 3) of cells. There were no significant differences in nuclear area based on location of cells within the EF. Left *n* = 82, middle *n* = 82, right *n* = 81.

Next, we measured whether applying an electrical current to cells grown on either of these scaffolds would change the morphology of the cells. Interestingly, we found there to be no statistically significant difference between stimulated and non-stimulated cells grown on different fiber alignments (Figure 6). Although not statistically significant, in general, ES did increase major and minor axis lengths (Figure 6b,c) and consequently, the nuclear area also increased (Figure 6a). For flat controls, there was a statistically significant increase in the major nuclear axis (Figure 6b—Flat), which most likely contributed to the general increase in nuclear area. The position of cells in relation to the EF did not appear to effect nuclear shape (Figure 7).

**Figure 8:**
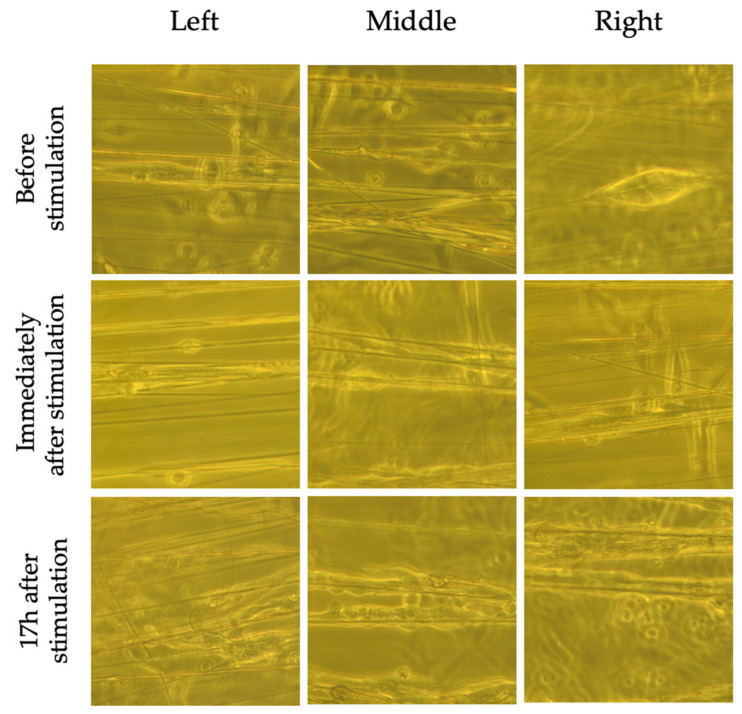
Bright field images of non-stimulated SH-SY5Y cells on aligned fibers before, immediately following and 17 h after ES. Cells near the electrode (left and right side as per Figure 3) do not appear to have changes in morphology or detach.

**Figure 9:**
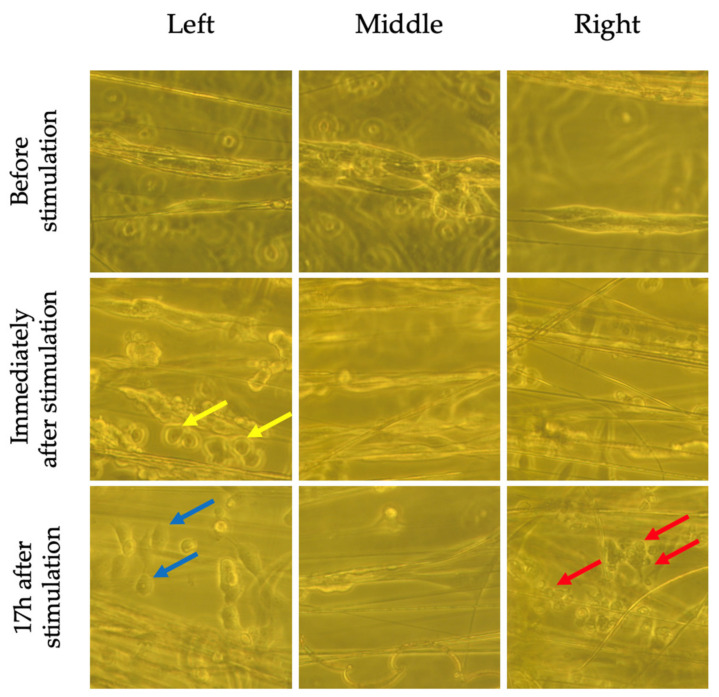
Bright field images of stimulated SH-SY5Y cells on aligned fibers before, immediately following and 17 h after ES. Cells near the electrodes (left and right as per Figure 3) appear to have shortened (blue arrow), have begun detaching (yellow arrow), and some appear dead (red arrow). The cells in the middle do not appear to be affected.

Although nuclear morphology was not significantly altered by the EF, bright field images (Figure 8 and Figure 9) taken immediately following and 17 h after stimulation revealed shortening of cells that were located to the left and right of the EF compared to non-stimulated cells. There was also evidence of cell detachment and possibly cell death (Figure 9). These changes were not observed in non-stimulated cells, suggesting that these findings were a result of the EF and not the electrospun fibers.

### 2.5. Marginal Increases in BDNF Expression in Cells Grown on Perpendicular Scaffolds Located in the Middle of the EF

**Figure 10:**
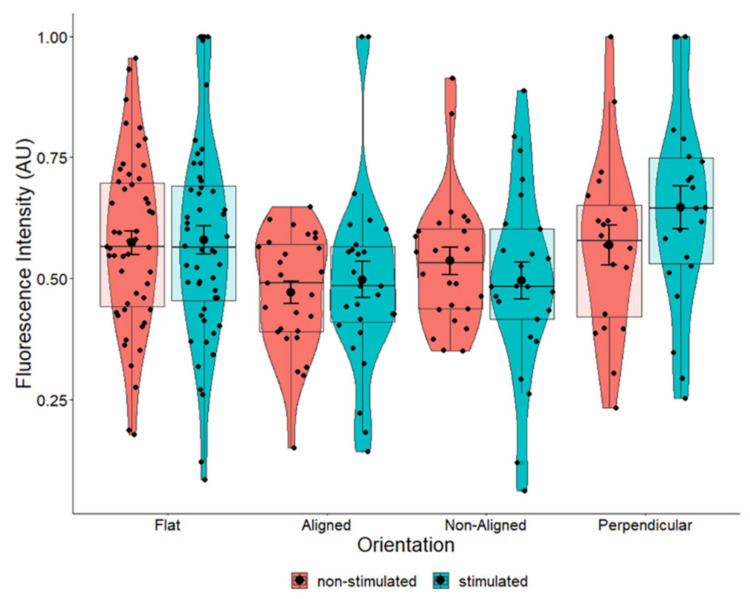
Pair-wise comparison of BDNF expression in SH-SY5Y cells with and without ES grown on flat cover slips, aligned fibers aligned with EF, non-aligned fibers, and aligned fibers perpendicular to the EF. One-way ANOVA for flat *p* = 0.857, aligned *p* = 0.551, non-aligned *p* = 0.407, and perpendicular *p* = 0.204. Flat *n* = 101, aligned *n* = 54, non-aligned *n* = 49, perpendicular *n* = 41.

When comparing stimulated to non-stimulated cells grown in the same fiber orientation, there were marginal increases in the average intensity of BDNF in cells grown on aligned and perpendicular fibers (Figure 10). We used a measure of effect size for analysis of variance (ANOVA) models termed Eta squared (η^2^) to explain the amount of variation afforded by the fibers in the total variation for the expression of BDNF, where values closer to 1 indicate a higher proportion of variance that can be explained by a given variable in the model. However, the marginal increases we observed were not statistically significant (Figure 10). The greatest increase in BDNF (*p* = 0.204) occurred in cells that were growing perpendicular to the electrical field (Figure 10). Eta squared values (Table 1) revealed that perpendicular fibers had a small to medium effect on the BDNF intensity and perpendicular fibers appeared to have the most influence on BDNF expression compared to other fiber orientations.

**Figure 11:**
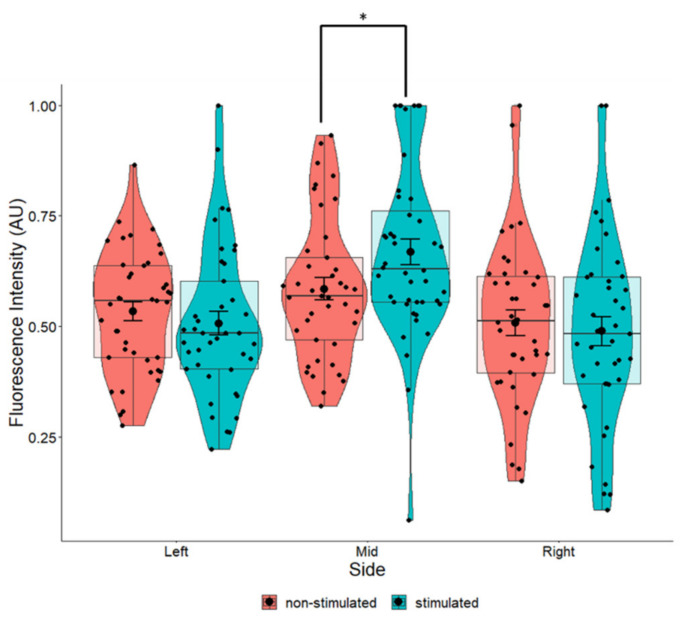
Pair-wise comparison of BDNF expression in SH-SY5Y cells with and without ES grown in relation to the position of the EF (based on Figure 3). One-way ANOVA *p* = 0.405 (**left**) *p* = 0.035 (**middle**) *p* = 0.672 (**right**). The most significant changes in BDNF expression were seen in cells found at the center of the EF. Left *n* = 82, middle *n* = 82, right *n* = 81. * *p* < 0.05.

The position of the cells relative to the EF did influence BDNF expression. Cells at the center of the EF had a statistically significant increase in BDNF expression (*p* = 0.034) compared to the non-stimulated cells regardless of fiber orientation (Figure 11). By evaluating the effects of both the fiber orientation and position of the cells in the EF, we found that cells in the middle of the field, especially those lying in a perpendicular orientation, had significantly higher expression of BDNF compared to non-stimulated cells (Table 2). Whist these parameters were the only ones that afforded a statistically significant increase in BDNF (Table 2), cells lying on aligned fibers in the middle of the EF displayed a high η^2^ value (η^2^ = 0.161), indicating that these parameters also had a large effect on BDNF expression.

### 2.6. Effects of EF and Fiber Orientation on Nuclear Morphology

**Figure 12:**
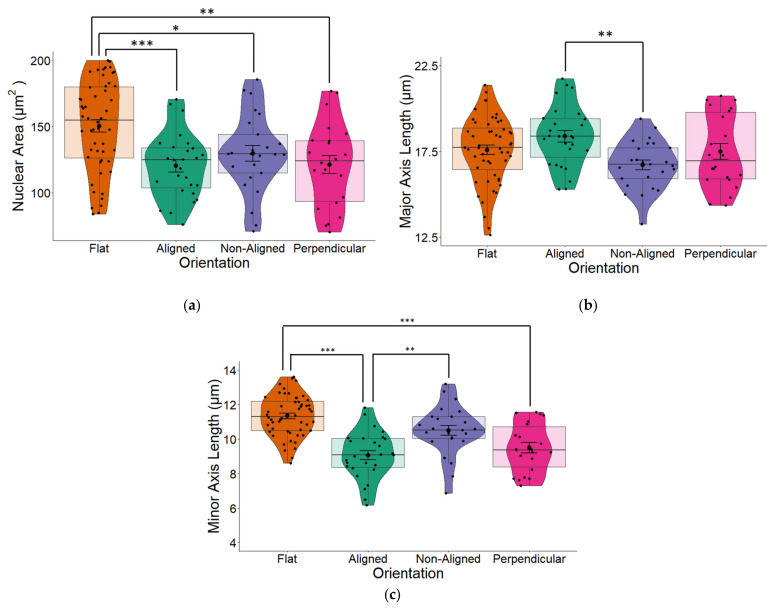
(**a**) The nuclear area of human SH-SY5Y cells grown on aligned or non-aligned scaffolds following ES. To determine whether ES had an additive effect on nuclear morphology, the cells were stimulated for 3 h using a 100 mV/mm direct current (DC) EF. The surface area of the nucleus was measured 17 h following stimulation and was found to be significantly altered (one-way ANOVA *p* < 0.001). Cells grown on aligned (*p* < 0.001) and non-aligned (*p* = 0.040) scaffolds had significantly reduced surface area compared to cells grown on the plain glass coverslips. Additionally, we also discovered that cells that were growing in the plane perpendicular to the electrical field also displayed smaller nuclear surface areas (*p* = 0.002) compared to cells growing on the glass coverslips. (**b**) Quantification of nuclear length along the major (long) axis of SH-SY5Y cells following ES. Stimulation of SH-SY5Y cells with a 100 mV/mm direct current for 3 h led to overall changes in nuclear morphology (One-way ANOVA *p* = 0.020). The length of the major axis of cells growing on aligned scaffolds was significantly different (*p* = 0.009) compared to cells growing on non-aligned scaffolds. (**c**) Quantification of nuclear length along the minor (short) axis of SH-SY5Y cells following ES. The most profound changes following ES were observed along the minor axes of cells growing on scaffolds. The minor axis of cells growing on aligned scaffolds showed the largest and most significant decreases compared to both control (*p* < 0.001) and non-aligned (*p* = 0.001) scaffolds. Decreases were also observed between perpendicular and flat controls. * *p* < 0.05, ** *p* < 0.01, *** *p* < 0.001. Flat *n* = 52, aligned *n* = 27, non-aligned *n* = 25, perpendicular *n* = 22.

Pair-wise comparison between stimulated and non-stimulated cells (Figure 6), suggest that fiber orientation has a larger influence on nuclear morphology compared to EF, which display very minimal changes between stimulated and non-stimulated cells. Results summarized in Figure 12 reveal that ES imparts statistically significant effects on the nuclear area of cells grown on fibers in any orientation compared to flat control cells. Compared to results displayed in Figure 4, where no EF was applied, these results suggest that an EF imparts an effect on nuclear morphology. 

We observed no significant differences in the nuclear area of cells growing on fibers perpendicular or aligned with the EF. Given that cells grown on electrospun scaffolds should be similar, other than the fact that the cells are growing either on fibers that are aligned with or perpendicular to the direction of the EF (when no EF is applied, Figure 4), results displayed in Figure 12 suggests that the EF imparts an effect on nuclear morphology.

The resulting change in nuclear area between fiber orientations following ES may be due to changes in the major and minor axis. Whilst the major axis of cells growing on aligned fibers was marginally larger than that of the control cells (Figure 12b), the minor axes of cells growing on aligned and perpendicular fiber orientations were significantly smaller compared to control cells (*p* < 0.001; Figure 12c).

### 2.7. Effects of EF and Fiber Orientation on BDNF Expression

**Figure 13:**
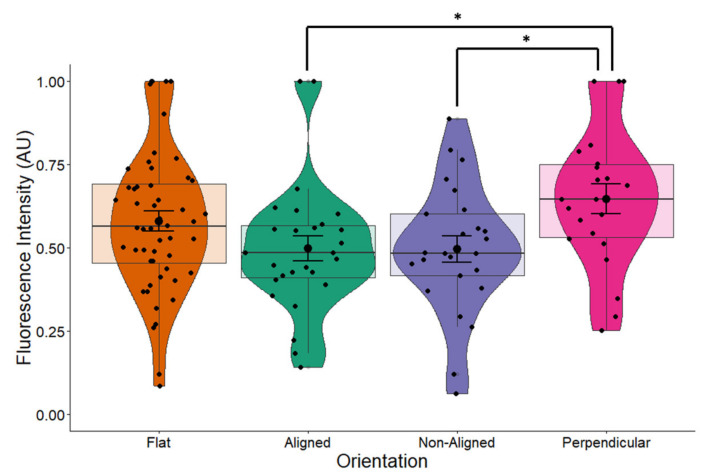
BDNF expression in ES cells. EFs have an additive effect to fiber orientation on the expression of BDNF. One-way ANOVA *p* = 0.02. Electrically stimulated cells growing on fibers, perpendicular to the EF had the highest expression of BDNF of the different orientations where there was a significant difference between cells growing on aligned and non-aligned fibers compared to cells growing on fibers perpendicular to the EF (Tukey post-hoc both *p* = 0.037). Flat *n* = 52, aligned *n* = 27, non-aligned *n* = 25, perpendicular *n* = 22. * *p* < 0.05.

The introduction of the EF had an additive effect to fiber orientation on the expression of BDNF (one-way ANOVA *p* = 0.02, Figure 13), especially for cells grown on perpendicular fibers. Comparison between stimulated and non-stimulated cells grown on flat surfaces does not result in changes in BDNF expression (Figure 10), indicating that ES alone does not affect BDNF expression. In non-stimulated cells, orientation of fibers had a marginal effect on BDNF expression (Figure 5, *p* = 0.055). However, the combination of an EF with fiber orientation appears to play a significant role on the expression of BDNF (Figure 13, ANOVA *p* = 0.02). This demonstrates a synergetic effect of orientation and ES on BDNF levels in SH-SY5Y cells.

## 3. Discussion

Neurons communicate with each other and with the rest of the body via electrical signals and are thus referred to as excitable cells. The generation of these signals and the transmission of information lead to changes in gene and protein expression. Peripheral nerve injuries resulting in damage to axons are a serious medical condition and, in many cases, complete functional recovery through regeneration is not possible due to hostile environments surrounding the damaged area, the loss of trophic support, as well as electrical signals from the cell body. The potential to use electrical signals to induce neuronal growth and support is an emerging field in biomedical engineering. Here, we describe how electrical stimulation delivered along nanofibrous scaffolds could modulate the expression of BDNF in SH-SY5Y human neuroblastoma cells. 

### 3.1. Fiber Orientation Influences Nuclear Morphology

The physical shape of tissue engineering scaffolds effect cell and nuclear morphology. Werner and colleagues have shown that convex-shaped substrates cause more nuclear deformation compared to concave-shaped substrates [33]. In the case of electrospun nanofibrous scaffolds, similar findings are evident when cells are grown on aligned and non-aligned fibers. Typically, cells take on an elongated morphology when grown on aligned scaffolds compared to non-aligned fibers [34,35]. Changes in the cytoskeleton of cells induce nuclear deformation [33,35,36]. Similar to cytoskeletal morphology, cells growing along fibers will commonly have elongated nuclear shapes compared to non-aligned and flat controls [35] as was seen in our results. The physical cue of the nanofiber scaffold confines the cell and nuclear shape, resulting in the more elongated nuclear morphology [36].

### 3.2. BDNF Expression Is Influenced by Fiber Orientation

Our results show that BDNF expression was lowered in aligned scaffolds compared to controls. As the topography of tissue engineering scaffolds effect cell and nuclear morphology, there are subsequent changes to gene and protein expression. The effects of scaffold morphology on stem cell differentiation have been extensively investigated. Ghollasi and colleagues found increased neural differentiation and expression of BDNF in human-induced pluripotent stem cells (iPSCs) grown on aligned compared to non-aligned fibers [37]. Schwann cells grown on aligned and non-aligned electrospun PCL fibers were found to express lower levels of BDNF compared to cells grown on non-coated surfaces [29]. Cells grown on aligned fibers had an even lower level of BDNF expression compared to those grown on non-aligned fibers [29], which are similar to our findings. Although no increases in neurotrophic factor expression were observed, Chew and colleagues reported that other genes related to Schwann cell maturation were changed. These changes included an upregulation of myelin-associated glycoprotein (MAG) and myelin protein zero (P0) and a down regulation of neural cell adhesion molecule 1 (NCAM-1) on aligned fibers only, suggesting that fiber alignment can affect gene expression [29]. Chew and colleagues [29] also found similar morphology between Schwann cells grown on aligned and non-aligned fibers to our results. Schwann cells grown on electrospun poly (3-hydroxybutyrate) (PHB) and poly (3-hydroxy butyrate-co-3-hydroxyvalerate) (PHBV) fibers were also found to have increased expression of BDNF when grown on aligned compared to non-aligned fibers [38]. The variation in results could be due to the diameter of the fibers or material compatibility, as PCL is a synthetic polymer, whereas PHB and PHV are considered biopolymers. Alternatively, the difference could also be ascribed to the pattern of the nanofibers. Masaeli and colleagues found that the addition of collagen into the scaffolds failed to significantly influence the expression of BDNF but significantly increased the expression of NGF and glial cell line-derived neurotrophic factor (GDNF) [38]. It is plausible that additional/other extracellular matrix molecules are required as chemical cues to affect the expression of BDNF. Some studies have reported that the alignment of electrospun nanofiber scaffolds (aligned or non-aligned), are important mediators of neurite extension or polarity [17]. Differentiated PC12 neurites were found to be longer when grown on aligned nanofiber scaffolds compared to non-aligned scaffolds [39] and rat Schwann cells (SCL 4.1/F7) were found to have multi-polar morphology on randomly aligned fibers compared to a bipolar morphology on aligned fibers [38]. These varying cell morphologies could be attributed to differences in cell behavior and gene expression due to the induction of mechanotransducive pathways.

### 3.3. EF Does Not Significantly Alter Nuclear Morphology

The application of a 100 mV/mm DC EF field for 3 h did not cause major changes in nuclear morphology of SH-SY5Y neuroblastoma cells. Many studies report changes in cell morphology in reaction to EFs. For example, Zhao and colleagues applied a 100 mV/mm DC EF to endothelial cells for 72 h, resulting in cells aligning perpendicular to the EF, with indications of cell alignment evident as early as 8 h [40]. These endothelial cells became more elongated compared to the non-stimulated cells [40]. Another study utilizing human mesenchymal stem cells similarly used a 100 mV/mm DC EF as well as 200 mV/mm. These cells also took on a more elongated morphology after exposure to EFs following 7–14 days of stimulation [41]. Further elongation was promoted by the use of the stronger EF [41]. Our results did not replicate these findings in relation to changes in nuclear morphology. This could be due to various factors, including the difference in cell type and the duration of stimulation, which in our study was 3 h. Zhao and colleagues found that elongation and re-orientation were both voltage and time dependent, where changes were not prominent at 3 h [41]. In addition, Zhao and colleagues performed their experiments in flat cell culture plates and therefore cannot account for the effect of electrospun fibers. Furthermore, although the aforementioned studies used similar strengths of EF, electrical configurations differed and could potentially account/explain the different outcomes. For example, Zhao and colleagues used capacitive ES [41], whereas in the present study, we employed direct stimulation. Furthermore, different currents may have also contributed to the observed variations. Koppes and colleagues used electrospun, aligned, and non-aligned PLLA scaffolds and 50 mV/mm DC EF and discovered that dorsal root ganglion (DRG) cell elongation was predominantly affected by electrospun scaffolds, whilst the EF enhanced elongation, but to a smaller extent [42]. The ES parameters used were different to the current study, being 50 mV/mm and 1 mA for 8 h [42].

### 3.4. Effects of ES on BDNF Levels

A comparison between stimulated and non-stimulated cells on corresponding fiber orientations does not reveal major differences in BDNF intensity. Although cells grown on perpendicular fibers display the largest increase between stimulated and non-stimulated cells, the results were not statistically significant. Considering cells that are positioned only in the middle of the EF (Figure 11), there is a statistically significant increase in BDNF expression between stimulated and non-stimulated cells, suggesting that the changes in BDNF expression does not occur near the electrodes. The electrical signal may produce redox products [43] or generate heat localized to the electrode [44], which may have a negative impact on cells (Figure 8 and Figure 9) and hence, BDNF expression. These effects appear to be localized to cells surrounding the electrode. Although nuclear morphology was significantly influenced by fiber orientation, it was not additionally affected by the EF (Figure 6 and Figure 7). This indicates that the changes in BDNF expression are most likely caused by the EF.

ES results in an influx of calcium ions entering the neuron via calcium channels that vary depending on the type of cell or the type of electrical stimulus. For example, in hippocampal neurons, the influx involves N-type calcium channels [45], whereas in Schwann cells, the influx involves T-type voltage-gated calcium ion channels [24]. The activation of these calcium ion channels appears to be important in regulating the expression of BDNF [46]. In Schwann cells, it has been reported that an electrically induced increase in the expression of BDNF involved activation of the mitogen-activated protein kinase (MAPK) and calcium/calmodulin-dependent protein kinase type IV (CAMKIV) pathways, both eventually increasing the phosphorylation of cAMP response element-binding protein (CREB) [24]. However, in the case of spinal cord neurons, increased expression of BDNF via ES appeared to involve activation of extracellular signal-regulated kinase (ERK) [46].

### 3.5. Effects of EF and Fiber Orientation on Nuclear Morphology

Considering stimulated and non-stimulated cells, our control cells, grown on glass coverslips, appeared to have more changes in nuclear morphology, evident in marginal increases in major and minor axes and hence nuclear area. This can be explained by considering that cells grown on flat tissue culture plates are not confined as cells grown on the electrospun fibers [36,47]. Stimulated cells grown on fibers had minimal changes to nuclear area compared to non-stimulated cells. Fiber orientation and ES can independently affect cellular morphology. However, Koppes and colleagues found that electrospun PLLA scaffolds had a larger effect over DRG elongation compared to 50 mV/mm DC EF stimulation for 8 h [42]. Our results appear to be similar in regard to changes in nuclear morphology.

### 3.6. Effects of EF and Fiber Orientation on BDNF Intensity

We explored the possibility that human SH-SY5Y neuroblastoma cells grown on nanofiber scaffolds could be enticed to upregulate their expression of BDNF following ES. We report that application of a low, 100 mV/mm direct current (DC) EF to SH-SY5Y cells over a period of 3 h resulted in increased BDNF protein expression up to 17 h following stimulation for cells grown on aligned fibers that were lying perpendicular to the direction of the EF. Our results demonstrate that the added combination of ES can further enhance the expression of BDNF. This is seen in cells grown in the perpendicular orientation.

Immunocytochemistry was utilized in this study as this method made it easy to correctly identify cells that were in the correct fiber and electric field orientation. When considering previous studies that have analyzed the effect of EF on BDNF expression utilizing mRNA levels, it is important to note that mRNA levels do not necessarily correlate with BDNF protein expression measured either by immunocytochemistry or Western blot [48]. In a previous study, increased mRNA expression of BDNF and NGF was observed in Schwann cells grown on electrospun fibers with and without ES. The cells were stimulated utilizing 100 mV/mm DC EF for 4 h and expression was analyzed after 24 and 48 h [49]. Huang and colleagues who used electrically active PCL electrospun nanofiber scaffolds coated with reduced graphene oxide found similar results to the present study where Schwann cells on both aligned and non-aligned fiber scaffolds either with or without ES were shown to secrete NGF [50]. However, their study demonstrated that cells grown on aligned fibers secreted higher levels of secreted NGF (measured by ELISA) compared to those grown on non-aligned fibers, irrespective of stimulation, whilst ES increased NGF secretion regardless of fiber alignment [50].

Future studies will need to delineate the mechanisms underlying these transcriptional and translational changes. It is hypothesized that the effect of the EF does not necessarily extend only to direct effects on the cytoskeleton and hence the nucleus, thereby inducing mechanotransductive effects, but also on ions and even protein concentration and distribution [51,52,53]. These in turn become chemical cues that influence cell behavior. One possible avenue of investigation would be to assess the role(s) of the mechanosensitive Piezo1 channels present in cell membranes [54]. These mechanosensitive ion channels open and close in response to mechanical stimuli exerted on the cell membrane and thereby allow ions and other solutes to flow across cell membranes. Piezo1 expressed in neurons has been shown to be an important mediator of the effects of ultrasound on neurons in vitro [55]. Ultrasound stimulation of mouse primary cortical neurons initiated calcium influx into the cells, resulting in increased nuclear c-Fos expression as well significantly increased levels of the proteins phospho-CaMKII and phospho-CREB [55]. Transcranial ultrasound has also been shown to increase levels of BDNF in vivo [56,57,58]. Based on these findings, we speculate that the increased expression of BDNF observed in our study may have been initiated by the ‘stretching’ and activation of Piezo1 mechanosensitive channels.

Alternatively, fiber alignment can induce changes in gene expression through nuclear mechanotransduction. Possible mechanisms underlying such nuclear mechanotransduction changes could include changes to chromatin modification or the degree of DNA packaging [14]. Varying post translational modifications create heterochromatin structures whereby regions of DNA maybe packaged more openly and or more condensed [59]. The amount of condensed chromatin can influence the nuclear size or mechanical properties of the nucleus [59]. Results presented in our study show that the nuclear area of all cells grown on electrospun fibers were smaller, where cells on aligned and perpendicular fibers had more elongated nuclei compared to non-aligned and controls. Furthermore, stimulation of cells grown on aligned and perpendicular fibers had uniquely smaller minor axes compared to stimulated cells grown on non-aligned fibers and flat surfaces. This nuclear shape would have affected nuclear transduction. The perpendicular fibers displayed the greatest change in BDNF intensity following ES, suggesting that there was a combined effect of this particular fiber alignment within the EF. Furthermore, supporting a synergetic effect of both EF and fiber alignment, is the significant difference in BDNF expression observed between cells that were growing on the left, middle, and right of the EF.

As mentioned above, cells grown on tissue culture plates commonly align perpendicular to the EF [40,41,60] in order to limit the voltage drop across the cell membrane [61,62]. These changes are paralleled in cells that undergo perpendicular alignment when exposed to uniaxial stretch forces, where cells again opt to align in this position in order to minimize stretch/strain [63]. It is possible that cells already aligned perpendicular to the EF in our investigation were already positioned in their preferred alignment in the EF, whereas cells constricted on the aligned fibers were most likely unable to migrate into this preferred alignment. Cells grown on flat controls and non-aligned fibers may have had the freedom to migrate into this alignment, but time would be required to observe these changes compared to cells already positioned perpendicular to the EF. This could possibly explain why the combination of the EF on the perpendicular fibers was more prominent than the other orientations regarding BDNF expression.

Whilst results presented here demonstrate that both fiber alignment and ES may contribute to BDNF expression, the role of fiber thickness, stimulation intensity and duration, gene and protein expression patterns and time course of expression remain to be elucidated.

## 4. Materials and Methods

### 4.1. Preparation of Polycaprolactone (PCL) Nanofiber Scaffolds

To prepare nanofiber scaffolds, a 15% PCL/acetone polymer solution was prepared and placed in a 23G Terumo syringe (Livingstone International, Mascot NSW, Australia). The tip of the syringe was connected to a Van de Graaf generator, producing ~200 kV. The polymer solution was released at a rate of 1.1 mL/h using an Alaric (Cardinal Health) IVAC TIVA infusion pump (Seven Hills, NSW, Australia). Glass coverslips were clipped onto a collector rotated at 200 rpm and placed in front of the ground plate. The syringe tip was placed 9 cm away from the collector. The electrospinning process ran for 10 min for each scaffold. To create non-aligned scaffolds, the glass coverslip was rotated 90° every 1 min, whereas for aligned scaffolds, the glass coverslip was kept in place for the whole 10 min.

The glass coverslips with the scaffolds were placed into 6-well tissue culture plates. Each well had 80% (*v/v*) ethanol added and was incubated for 30 min to sterilize the scaffolds. Following sterilization, the wells were washed with 1× phosphate buffer saline (PBS) for 10 min, twice before adding media and cells.

### 4.2. Cell Culture

Human SH-SY5Y neuroblastoma cells (Cell Bank Australia, Westmead, NSW, Australia) were cultured in T25 flasks in complete culture medium consisting of 1:1 DMEM/HAMF12, 10% fetal bovine serum (GIBCO, Thermo Fisher Scientific, Scoresby VIC, Australia), 1% sodium bicarbonate (GIBCO), 1% Penicillin-Streptomycin (GIBCO), 1% sodium pyruvate, 1% non-essential amino acids, and 1% L-glutamine. The media was changed every 2–3 days. When the flask was confluent, the flask was briefly rinsed in 1× PBS and 0.025% Trypsin/EDTA (GIBCO) was added to the flask and incubated in for 3 min at 37 °C. Complete medium was added to the flask to neutralize the trypsin and the solution was collected and placed in a 15 mL tube. The tube was centrifuged at 40 RCF for 3 min and the supernatant was removed and discarded. The cells were resuspended in fresh media and 1 × 10^3^ cells were added to each well. Media was changed after 3 days, and after 5 days from seeding, the cells were electrically stimulated.

### 4.3. Electrical Stimulation

On the day of stimulation, the old media was removed and 2 mL of fresh media was added to each well. The cells were stimulated for 3 h using a 100 mV/mm direct current (DC) EF at 37 °C in 5% CO_2_. Currents of 20–27 µA were generated. Platinum electrodes attached to the lid of the 6-well plate and connected to a circuit controlled by an Arduino nano (Jaycar Electronics, Rydalmere, NSW, Australia) were used to deliver the stimulation. The current and voltage were measured at the commencement and end of the experiment using an Economy Autorange Multimeter with Non-Contact Voltage Sensor (Jaycar Electronics, Rydalmere, NSW, Australia; Cat No. QM1529). Cells were fixed 17 h following the end of stimulation. Bright field images were taken before, straight after and 17 h following stimulation.

### 4.4. Immunocytochemistry

The media was removed and discarded. The cells were washed twice with 1× PBS and placed in warmed (37 °C) 4% paraformaldehyde solution for 10 min at 37 °C. The paraformaldehyde was removed, and the cells were washed three times with 1× PBS. PBS containing 0.1% Triton-X was added at room temperature to permeabilize the cells. The solution was incubated for 15 min, following which, the cells were washed three times with 1× PBS and blocked using 1% bovine serum albumin for an hour at room temperature. The cells were incubated with the primary antibodies BDNF (1:200; ab108319; Abcam, Melbourne, VIC, Australia) and β-tubulin (1:1000; Cat # T7816; Sigma-Aldrich; North Ryde, NSW, Australia) overnight at 4 °C. The cells were washed with 1× PBS before adding the secondary antibodies Goat Anti-Rabbit IgG H&L Alexa Fluor^®^ 488 (Cat No. ab150077, RRID: AB_2630356, Abcam, Melbourne, VIC, Australia) (1:350) and Goat Anti-Mouse Alexa Fluor^®^ 594 (Cat No. ab150116, AB_2650601; Abcam, Melbourne, VIC, Australia) (1:350) at RT in the dark for 45 min. The cells were washed twice with Triton-X (0.05%) in PBS and incubated in DAPI (Cat No. D9542, Sigma Aldrich; North Ryde, NSW, Australia) for nuclear staining for 3 min. The cells were washed a final time with Triton-X (0.05%) in PBS briefly before coverslips were placed with DPX mountant (Cat No. 06522, Sigma-Aldrich). The slides were stored at 4 °C prior to image analysis.

## 5. Conclusions

We have found that cells grown on aligned fibers that were positioned perpendicular to the EF direction had the greatest expression of BDNF following stimulation. Furthermore, an increase in BDNF was also observed in cells that were situated in the middle of the EF. The properties of these fibers most likely influence cell and nuclear morphology, further promoting the influence of ES on the expression of BDNF.

## Figures and Tables

**Table 1:** Eta squared (η^2^) of the intensity of BDNF between non-stimulated and stimulated cells on different fiber orientations. Perpendicular fibers appear to exert the largest effect compared to other orientations. However, a value of 0.040 suggests a small (0.01) to medium (0.06) effect on BDNF intensity.

Orientation	Eta Squared (η^2^)
Flat	0
Aligned	0.007
Non-Aligned	0.015
Perpendicular	0.040

**Table 2:** Comparison of stimulated and non-stimulated cells growing on different fiber orientations and positions in the EF. Cells growing on aligned fibers perpendicular to and in the middle of the EF demonstrate statistically significant changes in the expression levels of BDNF. These cells have an η^2^ of 0.488, suggesting a large effect on the expression of BDNF. Cells lying in the middle on aligned fibers and those lying on the left but perpendicular to the EF, an η^2^ of 0.161 and 0.166, respectively, suggesting a large effect of EF on the expression of BDNF. η^2^ 0.01 = small effect size; 0.06 = medium; 0.14 and higher = large effect size.

Side	Orientation	One-Way ANOVA	η^2^
Left	Aligned	0.907	0.001
	Flat	0.996	0
	Non-Aligned	0.740	0.008
	Perpendicular	0.148	0.166
Mid	Aligned	0.099	0.161
	Flat	0.373	0.024
	Non-Aligned	0.643	0.015
	Perpendicular	0.004	0.488
Right	Aligned	0.531	0.025
	Flat	0.641	0.007
	Non-Aligned	0.384	0.055
	Perpendicular	0.477	0.047

## Data Availability

Data is contained within the article.

## References

[1] Carvalho C.R., Oliveira J.M., Reis R.L. (2019). Modern Trends for Peripheral Nerve Repair and Regeneration: Beyond the Hollow Nerve Guidance Conduit. Front. Bioeng. Biotechnol..

[2] Jessen K.R., Mirsky R., Lloyd A.C. (2015). Schwann Cells: Development and Role in Nerve Repair. Cold Spring Harb. Perspect. Biol..

[3] Siemionow M., Brzezicki G. (2009). Chapter 8 Current Techniques and Concepts in Peripheral Nerve Repair. Int. Rev. Neurobiol..

[4] Muheremu A., Ao Q. (2015). Past, Present, and Future of Nerve Conduits in the Treatment of Peripheral Nerve Injury. BioMed Res. Int..

[5] Rinker B., Vyas K.S. (2014). Clinical Applications of Autografts, Conduits, and Allografts in Repair of Nerve Defects in the Hand. Clin. Plast. Surg..

[6] Wang E.W., Zhang J., Huang J.H. (2015). Repairing peripheral nerve injury using tissue engineering techniques. Neural. Regen. Res..

[7] Tada K., Nakada M., Matsuta M., Yamauchi D., Ikeda K., Tsuchiya H. (2020). Long-Term Outcomes of Donor Site Morbidity After Sural Nerve Graft Harvesting. J. Hand Surg. Glob. Online.

[8] Lin M.Y., Manzano G., Gupta R. (2013). Nerve Allografts and Conduits in Peripheral Nerve Repair. Hand Clin..

[9] Moore A.M., MacEwan M., Santosa K.B., Chenard K.E., Ray W.Z., Hunter D.A., Mackinnon S.E., Johnson P.J. (2011). Acellular nerve allografts in peripheral nerve regeneration: A comparative study. Muscle Nerve.

[10] Lasso J.M., Deleyto E. (2017). Current Status in Peripheral Nerve Xenotransplantation. Xenotransplantation—New Insights.

[11] Muzzio N., Moya S., Romero G. (2021). Multifunctional Scaffolds and Synergistic Strategies in Tissue Engineering and Regenerative Medicine. Pharmaceutics.

[12] Engler A.J., Sen S., Sweeney H.L., Discher D.E. (2006). Matrix Elasticity Directs Stem Cell Lineage Specification. Cell.

[13] Lee S., Kumar S. (2016). Actomyosin stress fiber mechanosensing in 2D and 3D. F1000Research.

[14] Maurer M., Lammerding J. (2019). The Driving Force: Nuclear Mechanotransduction in Cellular Function, Fate, and Disease. Annu. Rev. Biomed. Eng..

[15] Aloe L., Rocco M., Balzamino B., Micera A. (2015). Nerve Growth Factor: A Focus on Neuroscience and Therapy. Curr. Neuropharmacol..

[16] Bathina S., Das U.N. (2015). Brain-derived neurotrophic factor and its clinical implications. Arch. Med. Sci..

[17] Tian L., Prabhakaran M.P., Hu J., Chen M., Besenbacher F., Ramakrishna S. (2015). Coaxial electrospun poly(lactic acid)/silk fibroin nanofibers incorporated with nerve growth factor support the differentiation of neuronal stem cells. RSC Adv..

[18] Ramku E., Ramku R., Spanca D., Zhjeqi V. (2017). Functional Pattern of Increasing Concentrations of Brain-Derived Neurotrophic Factor in Spiral Ganglion: Implications for Research on Cochlear Implants. Open Access Maced. J. Med. Sci..

[19] Shi R., Borgens R.B. (1995). Three-dimensional gradients of voltage during development of the nervous system as invisible coordinates for the establishment of embryonic pattern. Dev. Dyn..

[20] Messerli M.A., Graham D.M. (2011). Extracellular Electrical Fields Direct Wound Healing and Regeneration. Biol. Bull..

[21] Ju C., Park E., Kim T., Kim T., Kang M., Lee K.S., Park S.M. (2020). Effectiveness of electrical stimulation on nerve regeneration after crush injury: Comparison between invasive and non-invasive stimulation. PLoS ONE.

[22] Gordon T. (2016). Electrical Stimulation to Enhance Axon Regeneration After Peripheral Nerve Injuries in Animal Models and Humans. Neurotherapeutics.

[23] Zhu R., Sun Z., Li C., Ramakrishna S., Chiu K., He L. (2019). Electrical stimulation affects neural stem cell fate and function in vitro. Exp. Neurol..

[24] Luo B., Huang J., Lu L., Hu X., Luo Z., Li M. (2014). Electrically induced brain-derived neurotrophic factor release from schwann cells. J. Neurosci. Res..

[25] Huang J., Ye Z., Hu X., Lu L., Luo Z. (2010). Electrical stimulation induces calcium-dependent release of NGF from cultured Schwann cells. Glia.

[26] Yang F., Murugan R., Wang S., Ramakrishna S. (2005). Electrospinning of nano/micro scale poly(l-lactic acid) aligned fibers and their potential in neural tissue engineering. Biomaterials.

[27] Kim J., Kim H.N., Lim K.T., Kim Y., Seonwoo H., Park S.H., Lim H.J., Kim D.H., Suh K.Y., Choung P.H. (2013). Designing nanotopographical density of extracellular matrix for controlled morphology and function of human mesenchymal stem cells. Sci. Rep..

[28] Christopherson G.T., Song H., Mao H.-Q. (2009). The influence of fiber diameter of electrospun substrates on neural stem cell differentiation and proliferation. Biomaterials.

[29] Chew S.Y., Mi R., Hoke A., Leong K.W. (2008). The effect of the alignment of electrospun fibrous scaffolds on Schwann cell maturation. Biomaterials.

[30] Cao H., Mchugh K., Chew S.Y., Anderson J.M. (2010). The topographical effect of electrospun nanofibrous scaffolds on thein vivoandin vitroforeign body reaction. J. Biomed. Mater. Res. Part A.

[31] Stirling D.R., Swain-Bowden M.J., Lucas A.M., Carpenter A.E., Cimini B.A., Goodman A. (2021). CellProfiler 4: Improvements in speed, utility and usability. BMC Bioinform..

[32] (2020). Does SPSS Offer Tukey-Kramer Post-Hoc Tests?. www.ibm.com.

[33] Werner M., Blanquer S.B., Haimi S.P., Korus G., Dunlop J.W., Duda G.N., Grijpma D.W., Petersen A. (2017). Cell Migration: Surface Curvature Differentially Regulates Stem Cell Migration and Differentiation via Altered Attachment Morphology and Nuclear Deformation. Adv. Sci..

[34] Arancibia S., Silhol M., Mouliere F., Meffre J., Höllinger I., Maurice T., Tapia-Arancibia L. (2008). Protective effect of BDNF against beta-amyloid induced neurotoxicity in vitro and in vivo in rats. Neurobiol. Dis..

[35] Huang C.-Y., Hu K.-H., Wei Z.-H. (2016). Comparison of cell behavior on pva/pva-gelatin electrospun nanofibers with random and aligned configuration. Sci. Rep..

[36] Doolin M.T., Ornstein T.S., Stroka K.M. (2019). Nuclear Deformation in Response to Mechanical Confinement is Cell Type Dependent. Cells.

[37] Ghollasi M., Poormoghadam D. (2021). Enhanced neural differentiation of human-induced pluripotent stem cells on aligned laminin-functionalized polyethersulfone nanofibers; a comparison between aligned and random fibers on neurogenesis. J. Biomed. Mater. Res. Part A.

[38] Masaeli E., Morshed M., Nasr-Esfahani M.H., Sadri S., Hilderink J., van Apeldoorn A., van Blitterswijk C.A., Moroni L. (2013). Fabrication, Characterization and Cellular Compatibility of Poly(Hydroxy Alkanoate) Composite Nanofibrous Scaffolds for Nerve Tissue Engineering. PLoS ONE.

[39] Yao L., O’Brien N., Windebank A., Pandit A. (2009). Orienting neurite growth in electrospun fibrous neural conduits. J. Biomed. Mater. Res. Part B Appl. Biomater..

[40] Zhao M. (2003). Electrical stimulation directly induces pre-angiogenic responses in vascular endothelial cells by signaling through VEGF receptors. J. Cell Sci..

[41] Khaw J.S., Xue R., Cassidy N.J., Cartmell S.H. (2022). Electrical stimulation of titanium to promote stem cell orientation, elongation and osteogenesis. Acta Biomater..

[42] Koppes A.N., Zaccor N.W., Rivet C.J., Williams L.A., Piselli J.M., Gilbert R.J., Thompson D.M. (2014). Neurite outgrowth on electrospun PLLA fibers is enhanced by exogenous electrical stimulation. J. Neural. Eng..

[43] Meng S., Rouabhia M., Zhang Z. (2021). Electrical Stimulation and Cellular Behaviors in Electric Field in Biomedical Research. Materials.

[44] Meng S., Rouabhia M., Zhang Z., Gargiulo G. (2011). Electrical Stimulation in Tissue Regeneration. Applied Biomedical Engineering.

[45] Balkowiec A., Katz D.M. (2002). Cellular Mechanisms Regulating Activity-Dependent Release of Native Brain-Derived Neurotrophic Factor from Hippocampal Neurons. J. Neurosci..

[46] Wenjin W., Wenchao L., Hao Z., Feng L., Yan W., Wodong S., Xianqun F., Wenlong D. (2011). Electrical stimulation promotes BDNF expression in spinal cord neurons through Ca(2+)- and Erk-dependent signaling pathways. Cell Mol Neurobiol..

[47] Chen B., Co C., Ho C.-C. (2015). Cell shape dependent regulation of nuclear morphology. Biomater..

[48] Guo Y., Xiao P., Lei S., Deng F., Xiao G.G., Liu Y., Chen X., Li L., Wu S., Chen Y. (2008). How is mRNA expression predictive for protein expression? A correlation study on human circulating monocytes. Acta Biochim. Biophys. Sin..

[49] Zhao Y., Liang Y., Ding S., Zhang K., Mao H.-q., Yang Y. (2020). Application of conductive PPy/SF composite scaffold and electrical stimulation for neural tissue engineering. Biomaterials.

[50] Huang Z., Sun M., Li Y., Guo Z., Li H. (2021). Reduced graphene oxide-coated electrospun fibre: Effect of orientation, coverage and electrical stimulation on Schwann cells behavior. J. Mater. Chem. B.

[51] Ghasemi-Mobarakeh L., Prabhakaran M.P., Morshed M., Nasr-Esfahani M.H., Baharvand H., Kiani S., Al-Deyab S.S., Ramakrishna S. (2011). Application of conductive polymers, scaffolds and electrical stimulation for nerve tissue engineering. J. Tissue Eng. Regen. Med..

[52] Freeman J.A., Manis P.B., Snipes G.J., Mayes B.N., Samson P.C., Wikswo Jr J.P., Freeman D.B. (1985). Steady growth cone currents revealed by a novel circularly vibrating probe: A possible mechanism underlying neurite growth. J. Neurosci. Res..

[53] Kotwal A. (2001). Electrical stimulation alters protein adsorption and nerve cell interactions with electrically conducting biomaterials. Biomaterials.

[54] Ridone P., Vassalli M., Martinac B. (2019). Piezo1 mechanosensitive channels: What are they and why are they important. Biophys. Rev..

[55] Qiu Z., Guo J., Kala S., Zhu J., Xian Q., Qiu W., Li G., Zhu T., Meng L., Zhang R. (2019). The Mechanosensitive Ion Channel Piezo1 Significantly Mediates In Vitro Ultrasonic Stimulation of Neurons. iScience.

[56] Su W.S., Wu C.H., Chen S.F., Yang F.Y. (2017). Transcranial ultrasound stimulation promotes brain-derived neurotrophic factor and reduces apoptosis in a mouse model of traumatic brain injury. Brain Stimul..

[57] Tufail Y., Matyushov A., Baldwin N., Tauchmann M.L., Georges J., Yoshihiro A., Tillery S.I., Tyler W.J. (2010). Transcranial Puled Ultrasound Stimulates Intact Brain Circuits. Neuron.

[58] Chen C.-M., Wu C.-T., Yang T.-H., Liu S.-H., Yang F.-Y. (2018). Preventive Effect of Low Intensity Pulsed Ultrasound against Experimental Cerebral Ischemia/Reperfusion Injury via Apoptosis Reduction and Brain-derived Neurotrophic Factor Induction. Sci. Rep..

[59] Uhler C., Shivashankar G.V. (2017). Regulation of genome organization and gene expression by nuclear mechanotransduction. Nat. Rev. Mol. Cell Biol..

[60] Bunn S.J., Lai A., Li J. (2019). DC Electric Fields Induce Perpendicular Alignment and Enhanced Migration in Schwann Cell Cultures. Ann. Biomed. Eng..

[61] Banks T.A., Luckman P.S.B., Frith J.E., Cooper-White J.J. (2015). Effects of electric fields on human mesenchymal stem cell behaviour and morphology using a novel multichannel device. Integr. Biol..

[62] MCooper S., Keller R.E. (1984). Perpendicular orientation and directional migration of amphibian neural crest cells in dc electrical fields. Proc. Natl. Acad. Sci. USA.

[63] Li Y., Huang G., Zhang X., Wang L., Du Y., Lu T.J., Xu F. (2014). Engineering cell alignment in vitro. Biotechnol. Adv..

